# Clinical analysis and literature review of a case of ovarian clear cell carcinoma with PIK3CA gene mutation: A case report

**DOI:** 10.1097/MD.0000000000030666

**Published:** 2022-09-16

**Authors:** Abdulkarim Mohamed Farah, Shiyu Gu, Yan Jia

**Affiliations:** a Department of Obstetrics and Gynecology, The Second Hospital of Jilin University, Changchun, Jilin Province, China.

**Keywords:** clear cell ovary carcinoma, clinical application, PIK3CA, prognosis

## Abstract

Ovarian clear cell carcinoma (OCCC) is an uncommon malignant form of 5 subtypes of ovarian cancer, accounting for approximately 5% to 25% of all ovarian cancers. OCCC is usually diagnosed at a young age and an early stage. More than 50% of patients are associated with endometriosis. It shows less sensitivity to platinum-based chemotherapies, high recurrence, and poor prognosis, especially late. However, platinum-based chemotherapies remain the first-line treatment. Meanwhile, new treatment modalities have been explored, including immune checkpoint inhibitors and PI3K-AKT-mTOR pathway inhibitors.

**Patient concern:**

A 48-year-old Chinese woman, Gravida2 Para1, complained of irregular and painful vaginal bleeding for 4 months.

**Diagnosis:**

The patient was diagnosed with stage IC ovarian clear cell carcinoma that presented with a mutation of the phosphatidylinositol 4,5-bisphosphate 3-kinase alpha subunit (PIK3CA) gene.

**Intervention:**

We performed an early diagnosis and complete surgical resection of the tumor with platinum-based chemotherapy.

**Outcome:**

This patient with mutation of the PIK3CA gene was sensitive to platinum-based chemotherapy, showed a significant downwards trend in tumor markers, and was in good health within the year of follow-up.

**Lessons:**

This study described an OCCC case that presented with a PIK3CA mutation and was successfully managed with careful and complete resection of the tumor. This patient with mutation of the PIK3CA gene was sensitive to platinum-based chemotherapy, showed a significant downwards trend in tumor markers, and did not have recurrence after a year of follow-up, indicating a reasonably good prognosis. Therefore, surgery plus platinum drug chemotherapy is still the best strategy for OCCC treatment. In addition, it is recommended for such patients to undergo genetic testing as much as possible to predict the clinical treatment effect.

## 1. Introduction

Ovarian clear cell carcinoma (OCCC) is an uncommon malignant type of the 5 subtypes of ovarian cancers, with specific genetic and molecular features.^[1]^ It is the second leading cause of death in ovarian cancers. However, it is relatively rare, accounting for 5% to 25% of all ovarian cancers. It has a high risk of recurrence and poor prognosis due to resistance to platinum-based chemotherapy, especially when diagnosed late.^[2]^ Geographically, OCCC incidence varies. It accounts for 5% to 10% of all epithelial ovarian cancers in North America and 12% in Western countries; however, it seems to be highest in East Asia, with an estimated 25% to 30% and 10.3% to 11.6% of all epithelial ovarian cancers in Japan and Korea, respectively,^[3,4]^ particularly among young people (median age, 50.2–55.7 years).^[1]^

In contrast to other ovarian carcinomas, most individuals with OCCC are often diagnosed at an early age and an early stage of the disease, with mild to moderate elevation of serum CA125 levels.^[5]^ More than 50% of OCCC cases are associated with endometriosis, 3 times more likely to cause OCCC than non-endometriosis cases.^[6]^ Another study found endometriosis in approximately 75% of patients with OCCC.^[7]^ Other risk factors for OCCC include early menarche, late menopause, low birth rate, and less oral contraceptive use.^[8]^ The clinical features of OCCC patients include a unilateral sizeable pelvic mass with abdominal discomfort and edema. Additionally, they present with hypercalcemia and thromboembolic vascular symptoms due to the high production of parathyroid hormone and stimulation of stanniocalcin-1 signaling mediated by interleukin-6 (IL-6).^[9,10]^

Several studies have demonstrated altered genes in OCCC.^[11]^ The most frequent genetic alterations are the phosphatidylinositol 4,5-bisphosphate 3-kinase alpha subunit (PIK3CA) and AT-rich interactive domain-containing protein 1A (ARID1A) genes.^[12,13]^ PIK3CA and ARID1A mutations promote sustained production of IL-6, leading to the rapid growth of the tumor.^[14]^ PIK3CA is a member of the PI3K-AKT-mTOR signaling pathway, the dysregulation of which is strongly associated with the development of human epithelial cancers, including OCCC.^[15,16]^ When it is activated, PI3K-Akt modulates the expression of downstream genes, leading to inhibition of apoptosis and promotion of cell proliferation.^[17]^ Furthermore, PIK3CA mutation enhances PI3K activity and promotes cell cycle progression, survival, and motility.^[18]^ The primary treatment for patients with OCCC is a surgical approach or optimal cytoreductive surgery, followed by systemic chemotherapy. Platinum-based chemotherapies are still first-line drugs, although resistance increases, especially in the late stages. It is only 20% to 50% effective in OCCC patients. This has given OCCC a high recurrence rate and a poor prognosis.^[19–22]^ Treatment is thus limited, particularly for recurrent cases. New therapeutic approaches have seen immune checkpoint inhibitors such as anti-PD-1/PD-L1 antibodies, while anti-PI3K, Akt, or mTOR are still in clinical trials.^[23]^

Despite being associated with OCCC pathogenesis, studies suggest that PIK3CA mutation could indicate a good prognosis and improved overall survival of OCCC patients.^[24–26]^ Other studies have further stated that it is associated with early-stage OCCC and the absence of residual tumors after the first surgery.^[27]^ These findings, however, are still unresolved, as counter findings have emerged that seem to suggest otherwise.^[28,29]^ In this study, we present a case of an OCCC with PIK3CA mutation and discuss the clinical implications of the mutation on the patient’s prognosis and response to therapy.

## 2. Case presentation

This study assessed the case of a 48-year-old Chinese woman, G2P1, who was admitted to our hospital in November 2020 and was referred from her local hospital suspected of ovarian cancer with the complaint of an irregular and painful menstrual cycle. She reported less vaginal bleeding with dark red and clotted menstrual blood. She also presented with a pelvic mass. She had sought treatment at a local hospital 3 weeks before her admission. She underwent a laparoscopic right adnexectomy and left ovarian cyst excision at the local hospital, and pathology specimens were collected. The postoperative pathological report (47,388) was as follows: (right) clear cell carcinoma, (left) ovarian endometriosis cyst, (right) coronal cyst of the ovary, (right) no cancer metastasis of fallopian tube, and the cytological examination of ascites exfoliation revealed no cancer cells. The patient was thus diagnosed with ovarian cancer. The significant postsurgical clinical symptoms observed were abdominal distension, constipation, and insomnia, while her weight did not change significantly.

From history taken upon admission to our hospital, none of her family members had suffered from gynecological tumors or any other form of cancer. A routine gynecological exam indicated typical vulval growth, vaginal patency, a medium quantity of white discharge with no strange odor, and a soft and smooth cervix. The uterus measured 6.0*6.0*4.0 cm and was positioned anteriorly with significant mobility. In the bilateral adnexa region, there was no apparent mass or pain. The patient had no thromboembolic risk factors. Other gynecological examinations with a 3-dimensional color Doppler ultrasound revealed that the uterus was 6.2*5.8*4.5 cm in size and in an anterior position. It also indicated a clear uterine cavity with an intima thickness of 0.3 cm. The uterine wall echo was nonuniform, punctuated with a few strong echoes observed in the muscular layer. Hypoechoic nodules of 1.4*1.0 cm in size were observed in the posterior wall. No double ovaries or masses were observed in the adnexal areas, and examination with color Doppler flow imaging revealed no abnormal blood flow signal.

In conclusion, the ultrasound impression diagnosis was myometrium heteroechogenicity with uterine fibroids as a possible cause. Experts conducted histological examinations in the pathology department on tissue samples from the local hospital. The investigation revealed carcinoma infiltration with morphology consistent with clear cell carcinoma in the right ovary. Since the tumor ruptured during surgery, it was impossible to perform lymphadenectomy or greater omentectomy, which would have allowed accurate staging. The patient had only undergone adnexectomy of the affected side.

As treatment intervention, we administered 3 courses of treatment combinations chemotherapy and scheduled a second operation considering the high recurrence rate and uncomplicated metastasis of this cancer. Informed consent needed to perform this treatment was freely obtained from the patient and her family members. Upon screening, no contraindications to chemotherapy were found. Therefore, the patient was given 3 courses of paclitaxel + carboplatin preoperative chemotherapy for her malignant tumor. There were no apparent complaints of discomfort or side effects, such as bone marrow suppression and impaired immune function, following this treatment. As shown in Table 1, the serum levels of tumor markers remained within the normal range during chemotherapy treatment.

**Table 1:** Serum levels of tumor markers before, during, and after chemotherapy treatment.

Tumor markers	On admission	After 1st chemotherapy	After 2nd chemotherapy	After 3rd chemotherapy
CA125 (U/mL)	21.10	10.60	7.60	7.10
CA199 (U/mL)	83.89	29.33	23.00	22.55
HE4 (pmol/L)	35.2	31.5	30.0	34.6

CA = cancer antigen, HE4 = human epididymis protein 4.

The patient’s condition was evaluated to determine the feasibility of surgical treatment upon completion of chemotherapy. The patient was subjected to a comprehensive preoperative examination. Pelvic enhanced computed tomography (CT) (multiphase) indicated that the uterine shape and size were standard; the cervix shape was total but of uneven internal density. A nodular soft tissue density shadow was observed at the bottom of the uterus, and part of the tissue protruded from the uterus. Additionally, the contrast-enhanced scan of the uterus and cervix indicated uneven enhancement. The oval-shaped low-density shadows were seen in the adnexal areas on both sides. On the right ovary, the size was approximately 17 mm × 15 mm with a CT value of about 18 Hu and no significant enhancement. On the left ovary, the measure was about 28 mm × 26 mm with a CT value of about 39 Hu, and a slightly heterogeneous enhancement was observed. This patient had an inadequate bladder capacity, and she had no enlarged lymph nodes in her pelvic cavity. No noticeable fluid density shadow was observed in utero-rectal depression. In conclusion, the results suggested no changes in the uterus, cervix, or bilateral adnexal areas.

In light of the above findings, surgical treatment was deemed feasible. Therefore, we performed a total abdominal hysterectomy, left adnexectomy, right adnexectomy, greater omentectomy, pelvic lymphadenectomy, and abdominal para-aortic lymphadenectomy under general anesthesia intubation on January 26, 2021. The postoperative pathological report (839489) was as follows: left appendage; no cancer observed, left ovary; a large number of hemosiderin cells and multinucleated giant cells and calcification in the greater omentum. Neither cancer nor adenomyosis, uterine leiomyoma, proliferative endometrium, or chronic cervicitis was found. No cancer metastasis was observed in the pelvic lymph plexuses or abdominal para-aortic lymph plexuses. The pathological results confirmed the diagnosis of malignant ovarian tumor (stage IC). Considering the patient’s condition, we gave 1 course, treatment combinations (paclitaxel 270 mg plus carboplatin 700 mg) chemotherapy, on the 7th day after surgery. The patient was followed regularly until recovery. The serum levels of tumor markers obtained after the fourth were as follows: CA125: 22.8 U/mL, CEA: 0.65 ng/mL, and CA72-4: 0.7. Overall, other tests conducted did not reveal any abnormalities.

### 2.1. Retrospective analysis of clinical information and genomic testing

Following successful case management, we reviewed the clinicopathological features of the patient to carry out further tests to identify the case’s molecular type. In doing so, we ascertained that the patient presented with a pelvic mass and a distended abdomen. We considered histological findings that revealed clear cell carcinoma and endometriosis cyst in the contralateral ovary. Informed consent was obtained from the above analysis, and genetic testing was ordered. Genetic testing was outsourced from Shanghai 3D Medical Laboratory on paraffin sections of the right ovary and peripheral blood collected from the patient. The test covered the whole exon region and some intron regions of 139 genes. The test results indicated no mutation in the BRCA1/2 and homologous recombination deficiency genes. Although mutations were observed in the genes PIK3CA, ARID1A, and SMARCA4, only PIK3CA was significantly altered. Therefore, it was concluded that the patient had a PIK3CA gene mutation at site P. E545exon10.

## 3. Discussion

In this study, we have described a case of OCCC that presented with the mutation of the PIK3CA gene and was successfully managed with an aggressive combination of chemotherapy and surgery. Similarly, Itamochi et al recommended complete removal of the tumor as therapy for OCCC, although this is difficult in advanced disease.^[30]^ The tumor was diagnosed early, consistent with published literature that acknowledges that 57% to 81% of OCCC cases are often diagnosed early stage I, while 19% to 22% are diagnosed at stage II.^[5]^

The prognosis of early OCCC was considerably higher than that of late OCCC. Based on the International Federation of Gynecology and Obstetrics stages, the 5-year overall survival rate and progression-free survival rate were 80% to 89% and 56% to 88% for stages I and II and reduced 52% to 25% for stages III and IV, respectively. In addition, the 3-year overall survival rates in stages I, II, III, and IV were 96%, 85%, 54% and 40%, respectively, and progression-free survival rates were 80%, 47%, 34%, and 30%, respectively.^[22,31]^ Early diagnosis has the advantage of allowing for a complete resection of the tumor before it spreads further and enables chemotherapy to eliminate any residual cancer cells effectively. To these effects, our patient responded well to 3 courses of platinum-based chemotherapy and surgery.

Platinum-based chemotherapies remain the first-line treatment for OCCC, despite the increase in resistance, especially for late-stage diagnosis.^[32,33]^ According to Itamochi et al,^[34]^ OCCC tumors have several characteristic features compared to other epithelial ovarian neoplasms, including a high incidence of stage I, a massive pelvic mass, and an increased risk of vascular thromboembolic sequelae and hypercalcemia. Additional variables contributing to drug resistance include diminished tumor growth, decreased drug deposition, faster drug elimination, and increased DNA repair capacity. Therefore, the rate of tumor recurrence among platinum-sensitive patients remains high, up to 70.5%, according to Ye et al.^[20]^ This is further complicated by reports stating that as many as 9% of platinum-sensitive patients become resistant after recurrence,^[19]^ while only 13.2% survive up to 5 years post-recurrence.^[33]^ After a year of follow-up, our patient had not developed recurrent malignancy. We believe this was due to the complete surgical resection of the tumor, which left no residual tumor cells behind because incomplete tumor resection leads to high recurrence rates. Even after post-recurrence debulking surgery, patients have the worst survival.^[33]^

Our patient had a significant PIK3CA gene mutation per genetic analysis, and the mutation site was P. E545exon10. ARID1A and SMARCA4 were also mutated but not substantially. However, the patient had no BRCA1/2 gene mutation. Similarly, previous studies have demonstrated that OCCC patients have less family history and rarely carry germline BRCA1/BRCA2 mutations. In addition, these tumors often have a deleterious p53 mutation and have fewer copy number variants.^[35]^ However, the immunohistochemical results of OCCC have shown that approximately 95% of patients have positive hepatocyte nuclear Factor 1, estrogen receptor, progesterone receptor, and Wilms tumor 1 are negative.^[36]^

PIK3CA is the catalytic subunit of the class I PI3-kinase enzyme part of the PI3K/AKT/mTOR pathway.^[37]^ It plays a role in cell growth, motility, survival, proliferation, protein synthesis, autophagy, transcription, and angiogenesis.^[24]^ Most studies agree that the primary molecular genetic alteration that activates the PI3K/AKT pathway in ovarian clear cell carcinoma is PIK3CA mutation. However, patients with mutant PIK3CA tumors had a slightly better overall prognosis than patients with wild-type PIK3CA tumors.^[25,38]^ It is estimated that approximately 40% to 66.7% of OCCC cases present with PIK3CA mutation.^[39–41]^ Another study found no significant relationship between the PI3KCA mutation and the histological characteristics of OCCC. As an outcome, PIK3CA expression has no impact on survival.^[28]^ Dysregulation of this pathway is strongly associated with OCCC pathogenesis and chemoresistance.^[42]^ PI3K/Akt activation promotes the active excretion of drug molecules from target cells,^[43]^ a process reinforced by PIK3CA mutation, which enhances PIK3 activity.^[44]^ The mutation is seen as early as endometriosis, which is considered the predecessor of OCCC.^[27]^ Despite this association, however, the clinical importance of this mutation is still debatable.

Inhibition of the PI3K/AKT/mTOR pathway is a new therapeutic target for OCCC. Some PI3K and mTOR inhibitors, such as everolimus, are in clinical trials.^[45,46]^ Given the inherent complexity of this pathway, targeting just one component of the pathway at a time has proven futile thus far; as a result, combination treatment with chemotherapies and antiangiogenic drugs has been adopted, and the results show improved prognosis.^[47,48]^ Despite its association with increased IL-6 secretion, which promotes OCCC development, PIK3CA mutation has, interestingly, been linked to good prognosis and improved overall survival in OCCC patients. Furthermore, ARID1A mutation that leads to activity loss is linked to poor prognosis and chemoresistance.^[49]^ Yoshida et al^[50]^ demonstrated a novel therapeutic target for HNF-1beta and PLK-Emi1, as well as signaling downregulation molecules that may be useful in patients with OCCC. IL-2 and IFN-alpha are effective treatments for patients with OCCC, alone or in conjunction with standard OCCC therapy. In another study, Mendoza et al^[51]^ showed that inhibiting the RasERK and PI3KmTORC1 signaling pathways reduced tumor formation in xenograft cancer models. In most aggressive OCCC cases, PIK3CA and ARID1A are significantly mutated,^[49]^ but our patient did not show significant ARID1A mutations. This could partly explain the chemosensitivity and the relatively good prognosis observed in the patient.

## 4. Conclusions

In summary, this study described an OCCC case that presented with a PIK3CA mutation and was successfully managed with careful and complete tumor resection. This patient with mutation of the PIK3CA gene was sensitive to platinum-based chemotherapy, showed a significant downwards trend in tumor markers, and did not have recurrence after a year of follow-up, indicating a reasonably good prognosis. Therefore, surgery plus platinum drug chemotherapy is still the best strategy for OCCC treatment. In addition, it is recommended for such patients to undergo genetic testing as much as possible to predict the clinical treatment effect.

## Author contributions

Conceptualization: Abdulkarim Mohamed Farah, Shiyu Gu, Yan Jia.

Resources: Shiyu Gu.

Supervision: Yan Jia. Validation: Yan Jia.

Visualization: Yan Jia.

Writing – original draft: Abdulkarim Mohamed Farah.

Writing – review & editing: Abdulkarim Mohamed Farah, Yan Jia.

All authors read and approved the final manuscript.
